# Effect of Biofermentation with Taxifolin on Physicochemical and Microbiological Properties of Cold-Smoked Pork Sausages

**DOI:** 10.17113/ftb.57.04.19.6250

**Published:** 2019-12

**Authors:** Sonata Gustiene, Gintare Zaborskiene, Anita Rokaityte, Reda Riešute

**Affiliations:** 1 Department of Food Safety and Quality, Lithuanian University of Health Sciences, Veterinary Academy, Tilzes St. 18, Kaunas LT-47181, Lithuania; 2 Food Institute, Kaunas University of Technology, Radvilenu St. 19, Kaunas LT-51180, Lithuania

**Keywords:** *Leuconostoc carnosum*, *Pediococcus pentosaceus*, *Staphylococcus xylosus*, biogenic amines

## Abstract

The aim of this work is to evaluate the effect of taxifolin on the physicochemical and microbiological properties of cold-smoked pork sausages produced using different commercial starter cultures with *Leuconostoc carnosum* and with a mixture of *Pediococcus pentosaceus* and *Staphylococcus xylosus*. Ultra performance liquid chromatography analysis demonstrated that after 181 days of storage total taxifolin content was the highest in samples with taxifolin and *L. carnosum* (60%), compared to the first day of storage. The sausages with taxifolin and the mixture of *P. pentosaceus* and *S. xylosus* (56%) followed next. Taxifolin improved the hygienic quality of sausages without significant effect on the growth of lactic acid bacteria. The accumulation of biogenic amines, including histamine and putrescine, was more effectively reduced in sausages inoculated with the taxifolin and *P. pentosaceus* and *S. xylosus* mixture. Using this mixture, the rate of lipolysis and lipid oxidation were effectively slowed down. Samples with taxifolin and *L. carnosum* showed the highest free radical scavenging activity on the first day of the study ((77.4±1.3) %) (p<0.05 in all samples). Mixtures containing taxifolin and starter cultures bound free radicals better than taxifolin alone. The colour parameters (L*, a* and b*) of preparations and final products were significantly influenced by taxifolin and starter cultures and storage time (p<0.05 in all samples).

## INTRODUCTION

The application of bioflavonoids as natural antioxidants for treatment of cold-smoked pork sausages produced using commercial starter cultures has not been discussed in the available literature. Taxifolin (also known as 3,5,7,3′,4′-pentahydroxy-flavanone or 2,3-dihydroquercetin) is a member of the group of flavanones (*1*). The satisfactorily pure taxifolin may be extracted from Siberian larch (*Larix sibirica* Ledeb.) and is also abundant in citrus fruits, grapes, olive oil and onions (*2*, *3*). As a common bioactive constituent of foods and herbs, taxifolin has been shown to exert a wide range of positive biochemical and pharmacological effects on human health. As a typical flavonoid, it inhibits free radical formation (*4*), influences the physical properties of lipids in biological membranes (*5*), has anti-inflammatory and analgesic properties (*6*), as well as cardioprotective and neuroprotective effects (*7*, *8*).

Taxifolin has many health-promoting effects, but is unstable under the light, with pH changes and during thermal treatment (*9*-*11*). Besides, little is known about the degradation behaviour of taxifolin during storage. Knowledge about the stability of taxifolin in food processing is important in order to predict the duration of physiological effects in food and beverages (*12*). European Regulation (EC) No 1129/2011 (*13*) reports the food additives authorized for use in Europe and their maximum permitted levels. On 13 December 2016, the EFSA NDA Panel adopted the Scientific Opinion on the safety of taxifolin as a novel food ingredient in non-alcoholic beverages, yoghurts, chocolate confectionery and food supplements pursuant to Regulation (EC) No 2015/2283 (*14*). The maximum proposed amounts of taxifolin are 0.02 g/L in non-alcoholic beverages, 0.02 g/kg in yoghurts and 0.07 g/kg in chocolate confectionery. The maximum proposed daily intake of taxifolin from food supplements is 100 mg/day (*15*). Scientific literature indicates the most suitable amount of taxifolin in meat products to be between 0.006 and 0.04%, depending on the fat content (*16*).

In the future, taxifolin could be used as a natural antioxidant and antimicrobial additive in the food industry (*17*), *e.g.* in cold-smoked fermented sausages with starter cultures due to the fact that taxifolin has been described as having antimicrobial (*18*) and radical scavenging activity (*19*), and a protective role in plants against pathogens (*20*). The addition of selected starter cultures has been reported to improve the safety of fermented sausages by restraining the development of undesired microorganisms, thus reducing the risk of pathogenic and spoilage bacteria, maintaining stability and shelf life, and enhancing the sensory characteristics of the meat products (*21*).

The aim of this work is to evaluate the effect of mixtures of taxifolin and different commercial starter cultures on physicochemical and microbiological properties of cold-smoked pork sausages in order to select the most suitable mixture regarding the safety and quality of sausages during storage.

## MATERIALS AND METHODS

### Preparation of taxifolin solution

Taxifolin (≥85%) was obtained from Sigma-Aldrich GmbH, Merck (Buchs, Switzerland). It was dissolved in several drops of ethanol (96%), diluted with double distilled water and added to the minced pork (0.517 mg/kg).

### Sausage production and sampling procedures

Eight different batches of pork sausages were manufactured using different techniques: three batches with the addition of different starter cultures in a proportion defined by the manufacturer (Chr. Hansen, Roskilde, Denmark), three batches with the addition of starter cultures and taxifolin, one batch with only taxifolin and one control batch without starter culture or taxifolin. The batches were prepared as follows: CR-1 with *Leuconostoc carnosum* starter culture, CR-2 with *Pediococcus pentosaceus* and *Staphylococcus xylosus*, CR-3 with *P. pentosaceus* in high quantity and *S. xylosus*, CR-4 with taxifolin and *L. carnosum*, CR-5 with taxifolin, *P. pentosaceus* and *S. xylosus*, CR-6 with taxifolin, *P. pentosaceus* in high quantity and *S. xylosus*, CR-7 with only taxifolin, and CR-8 (control) without taxifolin or starter culture.

The composition of pork sausages was as follows: whole pork muscle and back fat cuttings (80%) and raw pork ham (20%), purchased from a local butcher’s in Kedainiai, Lithuania, with the addition of the following ingredients (in g/kg): sodium chloride 25, lactose 20, dextrin 20, sodium caseinate 20, glucose 7, black pepper 1.5, white pepper 1 and sodium ascorbate 0.5, all purchased from Sauda, Garliava, Lithuania. The whole muscle cuts and raw ham were minced by electric meat grinder (Bosch MFW68660; Frankfurt, Germany) through a 13-mm diameter mincing plate and mixed together with the other ingredients for 3 min. The mixture was kept at 4 °C for 24 h in a refrigerator (Snaigė FR240-1101AA; Kaunas, Lithuania) and then stuffed into natural casings with a diameter of 45 mm and a length of 9 cm. Thin cold smoke was applied (Helia Smoker, Burbach, Germany) over a period of 24 h until the casings developed yellow to light brown colour. The sausages were fermented for 2 days at 15 °C and 85% relative humidity, then transferred into a ripening chamber where they were kept for 18 days at 10–12 °C and 75–80% relative humidity until the yield was 87% compared to the original meat mass. The cold-smoked sausages were then kept for 181 days at 15 °C and 75% relative humidity. Taxifolin stability, physicochemical properties and microbiological profile of sausages were analysed after 1, 33, 128 and 181 days of storage. The analyses were carried out in triplicates, and experiments were repeated twice.

### Determination of taxifolin in cold-smoked sausages using ultra performance liquid chromatography

All the reagents and standards of analytical grade, HPLC-grade acetonitrile, trifluoroacetic acid and taxifolin were from Sigma-Aldrich GmbH, Merck (Buchs, Switzerland). Deionized water was acquired from a Milli-Q purification system (Millipore Corporation, Bedford, MA, USA).

Sausage samples (1 g) were transferred into a 100-mL stoppered conical flask. Ethanol (96%, 100 mL) was added, and placed on a rotary shaker (Thermo Scientific MaxQ 4000 shaker; Thermo Fisher Scientific, Waltham, MA, USA) under agitation (300 rpm) for 30 min, then the mixture was filtered through filter paper (grade 601 A; Whatman, Maidstone, UK). The clear portion was kept in a freezer (Snaigė FR240-1101AA) for at least 20 min for the extraction of fat. The mixture was then centrifuged at 7500x*g* for 20 min at 4 °C (TJ-6 refrigerated centrifuge; Beckman Coulter, Palo Alto, CA, USA). The upper phase was filtered through a 0.2-µm pore-size syringe filter (Acrodisc LC13 PVDF; Sigma-Aldrich, Merck, Oakville, ON, Canada) and injected into the HPLC unit.

Ultra performance liquid chromatography (UPLC) was carried out with a Waters ACQUITY UPLC^®^ system consisting of binary solvent manager, auto sampler, column manager, and photometric diode array detector (Milford, MA, USA). The UPLC column was a 2.1 mm×100.0 mm Acquity UPLC BEH C18 (Waters) with 1.7 μm particles. The mobile phase consisted of 0.1% taxifolin in deionized water (A) and acetonitrile (B). The gradient was formed as follows: initially, the separation was started with 88% A and held for 1 min, then in 3 min A was decreased to 70%, after that within 3 min to 10%, and finally held at 10% for 1 min. Then the column was allowed to equilibrate for 2 min. The flow rate was 0.5 mL/min, and the injection volume was 1 µL. The detector was set in the 200–400 nm range. The chromatographic data were acquired and processed with Empower 3 software (*22*).

### Chemical analyses

The moisture content of cold-smoked pork sausage samples was determined according to ISO 1442:2000 (*23*). Approximately 1.0 g of sample was weighed in a moisture dish covered with a lid and the mass was recorded. The dish was then uncovered and placed in the oven (Universal Mechanical Oven UF 110 Plus; Memmert Frankfurt, Germany) at (102±2) °C for 2 h along with the lid. After drying, the dish was again covered with the lid and transferred to the desiccator to cool to room temperature. Then, it was carefully weighed and the process was repeated until the mass did not differ by more than 0.5 mg between measurements. Moisture content was then calculated from the following formula:
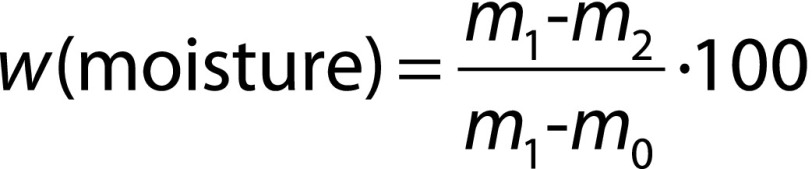
where *m*_0_ is the mass (in g) of the dish, rod and sand, *m*_1_ is the mass (in g) of the dish containing the test portion, rod and sand before drying, and *m*_2_ is the mass (in g) of the dish containing the test portion, rod and sand after drying.

The pH of sausages was measured according to the standard method ISO 2917:2002 (*24*) using a PP-15 professional pH meter (Sartorius, Goettingen, Germany).

For radical scavenging activity, the method used by Takao *et al.* (*25*) was adopted with suitable modifications from Kumarasamy *et al*. (*26*). The diluted sample (200 µL) was mixed with 800 µL of Tris-HCl buffer (100 mM, pH=7.4; Sigma-Aldrich, Merck, Goettingen, Germany). To this, 1 mL of 500 µM DPPH in ethanol (Sigma-Aldrich, Merck, Saint Louis, MO, USA) was added, the mixture was vortexed and absorbance was measured using Helios Gamma spectrometer (Thermo Scientific, London, UK) at 517 nm after 20 min of incubation in the dark. Percentage of radical scavenging activity was calculated as:

Radical scavenging activity=[(*A*_control_-*A*_extract_)/(*A*_control_)]·100 /2/

Acid value of the extracted lipids was determined according to ISO 660:2009 (*27*). A mass of 1 g of extracted lipids was dissolved with 50 mL neutral solvent solution (50 mL diethyl ether, 50 mL ethyl alcohol, and 1 mL 1% phenolphthalein solution (Sigma-Aldrich, Merck). The titration was carried out with 0.1 mol/L NaOH under constant shaking until the formed pink colour was persistent for 15 s. Acid value was calculated as follows:
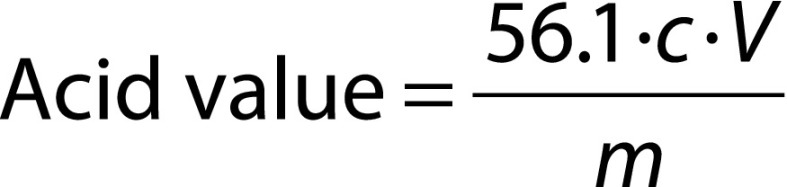
where 56.1 (in g/mol) is the molecular mass of KOH, *c* is the exact concentration (in mol/L) of the standard volumetric sodium or potassium hydroxide solution used, *V* is the volume (in mL) of standard volumetric sodium or potassium hydroxide solution used, and *m* is the mass (in g) of the sample.

Peroxide value of the studied lipids was determined according to ISO 3960:2017 (*28*). About 3.0 g fat were mixed with 50 mL of the solvent mixture (*V*(glacial acetic acid)/*V*(chloroform)=3:2; Sigma-Aldrich, Merck, Saint Louis, MO, USA), 1 mL freshly prepared saturated potassium iodide (Sigma-Aldrich, Saint Louis, USA) solution and 100 mL water. The titration was carried out with 0.01 mol/L sodium thiosulfate solution (Sigma-Aldrich, Merck, Saint Louis, MO, USA), using 1 mL starch solution and 0.1 g Thyodene™ indicator (Sigma-Aldrich, Merck, Saint Louis, MO, USA) until the blue colour disappeared. Peroxide value was calculated as follows:

Peroxide value= /4/

where *V* is the volume (in mL) of sodium thiosulfate solution used for the determination, *V*_0_ is the volume (in mL) of sodium thiosulfate standard solution used for the blank test, *c*_thiosulfate_ is the concentration (in mol/L) of the sodium thiosulfate solution, *m* is the mass (in g) of the sample, F is the factor of the 0.01 M sodium thiosulfate solution expressed in mmol of oxygen per kg of lipid.

### Microbial analysis

Samples of 10 g were taken at random for each batch, and aseptically weighed into a sterile stomacher bag with 90 mL of sterile buffered 0.1% (*m*/*V*) peptone water (Sigma-Aldrich, Saint Louis, USA) and homogenized for 1 min in a 400 Stomacher (Seward Ltd., Worthing, UK). Serial decimal dilutions were made, and lactic acid bacteria were determined by plate count on de Man, Rogosa and Sharpe agar (MRS; Oxoid, Basingstoke, UK) after incubation at 30 °C for 120 h. The number of yeast and mould colonies was determined by plate count on Dichloran Rose Bengal Chloramphenicol (DRBC) agar (Sigma-Aldrich, Merck, Saint Louis, MO, USA) after incubation at 25 °C for 120 h, and total count of mesophilic bacteria was determined on plate count agar (PCA, Sigma-Aldrich, Merck, Saint Louis, MO, USA) after incubation at 30 °C for 72 h in a thermostat. After incubation, colonies were counted according to ISO 7218:2007 (*29*). The microbiological data were transformed into the logarithm of the number of colony forming units (CFU/g).

### Biogenic amine content

A reversed-phase high-performance liquid chromatography (RP-HPLC) method was used for the quantitative analysis of biogenic amines tryptamine, phenylethylamine, putrescine, cadaverine, histamine, tyramine, spermidine and spermine. The whole cured sausage (edible parts) was cut into small pieces and mashed mechanically using a homogeniser (Moulinex Masterchef 20; Nieune, France). Biogenic amine content was extracted from a homogenized sample with 0.4 mol/L perchloric acid (Sigma-Aldrich, Merck, Saint Louis, MO, USA). The samples were derivatized for 45 min with dansyl chloride (5-dimethylaminonaphtalene-1-sulfonylchloride) solution in acetone (both Sigma-Aldrich, Merck, Saint Louis, MO, USA) at 40 °C using the modified method of Ben-Gigirey *et al.* (*30*). The samples were filtered through a 0.45 μm membrane filter (Millipore), and 10 μL of the sample were injected into chromatographic system (Agilent 1200 series; Waldbronn, Germany). The analysis was performed using LiChro column CART^®^ 95 125-4 (Merck, Darmstadt, Germany).

### Colour determination

Meat surface colour was measured using a reflectance spectrophotometer (Minolta CM-2002; Osaka, Japan). Parameters measured in the reflection mode were L*, a* and b* (corresponding to the brightness, redness and yellowness coordinates according to the CIE scale) (*31*).

### Statistical analysis of the data

The results are expressed as mean value±standard error of the mean. One-way analysis of variance (ANOVA) followed by Fisher’s least significant difference test, and the Dunnet test were applied when control group was present. Student’s *t*-test was used to determine average values of indicators, standard deviations and linear correlations. The correlation was considered reliable when p<0.05. SAS Studio v. 5.1 (*32*) served for data computation.

## RESULTS AND DISCUSSION

### Acidity of cold-smoked pork sausages

During storage, starter cultures in cold-smoked pork sausages affected the acidification of meat. The fermentation of carbohydrates causes the decrease of the active acidity (pH), which leads to the accumulation of organic acids, mainly lactic acid, during storage (*33*-*35*). The significant difference between the control and the samples with the added starter culture was detected before day 33 of the study (p<0.05 in all samples) (Table 1). We found a negative correlation between the low pH and high amount of lactic acid bacteria (R=-0.621, p<0.05). Throughout the study, significantly lower (p<0.05) pH values were found only of samples CR-3 and CR-6 than of control (CR-8). Taxifolin did not have significant effect on pH during storage. At the end of the study, the final pH value of cold-smoked pork sausages was around 5 (5.21 to 5.49) except of sample CR-4 (4.41). These data are in agreement with Berardo *et al.* (*36*), who claim that when using the Nordic sausage production technology, the final pH of the sausage is 5 or lower, while when using the Mediterranean technology, the final pH is between 5 and 6.

**Table 1:** Effect of taxifolin and commercial starter cultures on the pH of cold-smoked pork sausages during storage

Sample	*t*(storage)/day				
1	33		128	181
pH				
CR-1	(4.83±0.02)^a^		(4.93±0.02)^a^	(5.24±0.02)^a^	(5.36±0.01)^b^
CR-2	(4.85±0.01)^a^		(4.86±0.02)^a^	(5.31±0.03)^a^	(5.41±0.01)^a^
CR-3	(4.72±0.01)^a^		(4.82±0.01)^a^	(5.17±0.02)^b^	(5.23±0.03)^b^
CR-4	(4.81±0.02)^a^		(4.94±0.03)^a^	(5.25±0.01)^a^	(4.41±0.01)^a^
CR-5	(5.84±0.02)^a^		(4.91±0.02)^a^	(5.31±0.03)^a^	(5.43±0.02)^a^
CR-6	(4.72±0.03)^a^		(4.78±0.01)^a^	(5.07±0.01)^b^	(5.21±0.01)^b^
CR-7	(5.01±0.01)^b^		(5.11±0.04)^b^	(5.34±0.02)^a^	(5.49±0.02)^a^
CR-8	(4.98±0.02)^b^		(5.16±0.01)^b^	(5.29±0.02)^a^	(5.46±0.01)^a^

Results are expressed as mean value±standard deviation. Different letters in superscript indicate significant difference between the samples in the same row. CR-1=sausage sample fermented with *Leuconostoc carnosum* starter culture, CR-2=sausage fermented with *Pediococcus pentosaceus* and *Staphylococcus xylosus*, CR-3=sausage fermented with *P. pentosaceus* in high quantity and *S. xylosus*, CR-4=sausage with taxifolin and *L. carnosum*, CR-5=sausage with taxifolin, *P. pentosaceus* and *S. xylosus*, CR-6=sausage with taxifolin, *P. pentosaceus* in high quantity and *S. xylosus*, CR-7=sausage with taxifolin, and CR-8=sausage without taxifolin or starter culture (control)

### Microbiological profile of cold-smoked pork sausages

Table 2 shows the microbiological profile of cold-smoked pork sausages. The use of starter cultures increased (p<0.05) the population of lactic acid bacteria compared to the control sample (without starter culture). Our findings are similar to those reported by Kameník *et al.* (*37*), who observed that the lactic acid bacteria grow during the first days of fermentation and the population density peaks at 10^9^ cell/g. This is especially important because taxifolin does not inhibit lactic acid bacteria growth in the fermented sausages.

**Table 2:** Effect of taxifolin and commercial starter cultures on lactic acid bacteria, mesophilic bacteria, yeast and mould counts in cold-smoked pork sausages during storage

Microbiological profile	Sample	t(storage)/day			
1	33	52	147
*N*/(log CFU/g)			
Lactic acid bacteria	CR-1	(7.2±0.5)^b^	(6.0±0.2)^ab^	(6.3±0.4)^a^	(4.7±0.3)^a^
	CR-2	(7.0±0.3)^b^	(6.3±0.3)^ab^	(6.4±0.3)^a^	(4.4±0.4)^a^
	CR-3	(7.0±0.4)^ab^	(6.6±0.5)^b^	(6.7±0.4)^a^	(5.8±0.4)^a^
	CR-4	(7.3±0.4)^b^	(5.2±0.4)^ab^	(6.3±0.3)^a^	(4.3±0.3)^a^
	CR-5	(7.1±0.5)^b^	(6.4±0.3)^ab^	(6.9±0.4)^a^	(5.0±0.4)^a^
	CR-6	(6.3±0.3)^a^	(7.0±0.3)^b^	(6.9±0.2)^a^	(6.2±0.3)^a^
	CR-7	(6.2±0.4)^a^	(6.1±0.3)^ab^	(3.7±0.3)^b^	(3.0±0.2)^b^
	CR-8	(6.5±0.4)^a^	(5.7±0.3)^a^	(3.9±0.3)^b^	(3.3±0.3)^b^
Total count of yeasts and moulds	CR-1	(3.0±0.1)^a^	(3.1±0.2)^ab^	(3.2±0.3)^b^	(3.3±0.5)^b^
	CR-2	(2.0±0.2)^b^	(2.7±0.2)^a^	(3.5±0.2)^ab^	(4.0±0.3)^ab^
	CR-3	(2.6±0.2)^ab^	(2.9±0.3)^a^	(3.3±0.3)^ab^	(4.0±0.3)^ab^
	CR-4	(3.3±0.1)^a^	(3.2±0.2)^ab^	(3.2±0.3)^b^	(3.2±0.4)^b^
	CR-5	(2.2±0.3)^b^	(2.7±0.3)^a^	(3.8±0.2)^ab^	(4.2±0.4)^a^
	CR-6	(2.9±0.2)^ab^	(3.7±0.3)^ab^	(3.9±0.2)^ab^	(4.1±0.3)^ab^
	CR-7	(3.0±0.2)^ab^	(3.9±0.2)^b^	(3.9±0.3)^a^	(4.0±0.3)^a^
	CR-8	(3.9±0.3)^a^	(4.1±0.2)^b^	(4.3±0.4)^a^	(4.4±0.4)^a^
Total count of mesophilic bacteria	CR-1	(7.4±0.3)^a^	(6.9±0.3)^ab^	(6.3±0.3)^a^	(4.7±0.3^a^
	CR-2	(7.5±0.3)^a^	(7.0±0.5)^ab^	(6.5±0.2)^a^	(4.5±0.4)^a^
	CR-3	(8.2±0.4)^a^	(7.6±0.2)^b^	(6.4±0.3)^a^	(5.8±0.3)^b^
	CR-4	(7.4±0.5)^a^	(6.9±0.4)^a^	(4.8±0.4)^b^	(4.0±0.2)^a^
	CR-5	(7.5±0.4)^a^	(7.7±0.3)^b^	(6.8±0.2)^a^	(6.0±0.4)^b^
	CR-6	(8.1±0.3)^a^	(8.0±0.3)^b^	(7.0±0.4)^a^	(6.1±0.3)^b^
	CR-7	(8.2±0.3)^a^	(6.9±0.4)^ab^	(6.2±0.3)^a^	(3.0±0.1)^a^
	CR-8	(7.0±0.2)^a^	(6.2±0.3)^a^	(6.4±0.4)^a^	(3.3±0.2)^a^

Results are expressed as mean value±standard deviation. Different letters in superscript indicate significant differences between the samples in the same row. Sample abbreviations are given in Table 1

We also evaluated the yeast and mould counts. Taxifolin had an inhibitory effect on mould and yeast in CR-2 and CR-5 samples up to 33 days, and in CR-1 and CR-4 mixtures between days 33 and 181, compared to the control sample (p<0.05). Malterud *et al.* (*38*) studied the antimicrobial properties of flavonoids from *Salix caprea* and found that taxifolin was effective against bacteria and fungi. Taxifolin has also been identified in *Populus tremuloides* black galls, which are a type of plant tumours found in trees resistant to fungal infections (*39*).

### Accumulation of biogenic amines in cold-smoked pork sausages

Biogenic amines can be detected in raw materials and food products that are formed during metabolic processes. The main biogenic amines produced in the sausage during fermentation are putrescin, cadaverine and tyramine (*40*). In our study, sausages mainly contained (in mg/kg): putrescine 51.1-86.7, tyramine 15.0-56.6, cadaverine 19.2-34.6 and spermine 1.1-8.8 (data not shown). After 33 days, CR-5 batch had significantly (p<0.05) lower mass fraction of histamine (2.0 mg/kg) and putrescine (18.0 mg/kg) than other sausage batches (8.2-19.4 and 37.1-68.5 mg/kg respectively). A significantly (p<0.05) higher mass fraction of tyramine was observed in CR-2 than in CR-5 batch ((35.3±3.1) and (21.6±4.3) mg/kg respectively). The total biogenic amine content in CR-5 sausages was 25 and 49% lower (p<0.05) than that in CR-2 and CR-8 (control) sausages. CR-4 batch had a 31% lower biogenic amine content than CR-1, which had the highest total biogenic amine content ((204.5±11.6) mg/kg).

### Stability of taxifolin in cold-smoked pork sausages

A far more objective view of changes in taxifolin content in the cold-smoked pork sausages can be obtained after recalculation of the taxifolin content on a dry mass basis. This recalculation eliminates the effect of variable water content during production. After 181 days of storage, total taxifolin content was the highest in CR-4 batch (0.027 mg/kg, followed by CR-6 batch (0.025 mg/kg) (p<0.05), while the sausages treated only with taxifolin (CR-7) retained only 0.012 mg/kg (Table 3). UPLC analysis demonstrated that taxifolin was more stable in samples fermented with commercial starter cultures, which could be related to a stronger acidification during the first 33 days.

**Table 3:** Taxifolin content of cold-smoked pork sausages on dry mass basis during storage

Sample	*t*(storage)/day			
1	33	128	181
*w*(taxifolin)/(mg/kg)			
CR-4	(0.045±0.004)^a2^	(0.041±0.003)^a2^	(0.033±0.001)^b2^	(0.027±0.002)^c2^
CR-5	(0.043±0.003)^a23^	(0.038±0.002)^a2^	(0.029±0.002)^b2^	(0.022±0.003)^c2^
CR-6	(0.041±0.003)^a23^	(0.038±0.003)^a2^	(0.030±0.001)^b2^	(0.025±0.001)^c2^
CR-7	(0.039±0.004)^a3^	(0.030±0.001)^b3^	(0.022±0.002)^c3^	(0.012±0.001)^d3^

Results are expressed as mean value±standard deviation. Different letters in superscript indicate significant differences between the samples in the same row and different numbers in superscript indicate significant differences between the samples in the same column. Sample abbreviations are given in Table 1

### Antioxidant properties of cold-smoked pork sausages

The effect of taxifolin on antioxidant properties of cold-smoked pork sausages during storage is shown in Table 4. CR-4 sample had the highest free radical scavenging activity ((77.4±1.3) %) on the first day of the study, followed by CR-5, CR-6 and CR-7 ((76.9±0.8), (76.7±0.7) and (73.0±0.4) %, respectively) (p<0.05). Mixtures containing taxifolin with starter cultures bind free radicals better than taxifolin alone. On the last day of the study (day 181), the highest antioxidant activity was detected in CR-4, CR-5 and CR-6 batches ((55.6±0.5), (51.1±0.3) and (54.5±0.4) %, respectively). Kim *et al.* (*41*) stated that taxifolin was more effective antioxidant than BHA and α-tocopherol. Taxifolin has two aromatic rings that have two phenolic groups (–OH) at the *meta* and *para* positions. It is this arrangement that is the chief determinant of the strong antioxidant capacity of phenolic compounds (*42*).

**Table 4:** Effect of taxifolin and commercial starter cultures on antioxidant activity of cold-smoked pork sausages during storage

	Sample	*t*(storage)/day			
1	33	128	181
Scavenging activity/%	CR-1	(64.6±0.5)^a^	(57.4±0.8)^a^	(52.6±0.6)^a^	(43.3±0.5)^a^
	CR-2	(62.6±0.7)^a^	(58.8±0.4)^a^	(50.3±0.6)^a^	(44.6±0.5)^a^
	CR-3	(64.2±0.3)^a^	(57.1±0.4)^a^	(51.1±0.4)^a^	(42.8±0.6)^a^
	CR-4	(77.4±0.3)^b^	(74.3±0.7)^b^	(65.2±0.6)^b^	(55.6±0.5)^b^
	CR-5	(76.9±0.8)^b^	(73.6±0.4)^b^	(60.4±0.6)^b^	(51.1±0.3)^b^
	CR-6	(76.7±0.7)^b^	(73.2±0.4)^b^	(64.0±0.4)^b^	(54.5±0.4)^b^
	CR-7	(73.0±0.4)^b^	(68.1±0.4)^b^	(53.5±0.4)^a^	(47.1±0.7)^a^
	CR-8	(63.3±0.5)^a^	(57.7±0.5)^a^	(50.9±0.4)^a^	(44.7±0.6)^a^
Acid value/(mg /kg)	CR-1	(3.7±0.2)^a^	(7.1±0.3)^a^	(13.4±0.4)^a^	(24.2±0.3)^a^
	CR-2	(4.2±0.2)^a^	(6.8±0.3)^a^	(14.3±0.3)^a^	(26.5±0.3)^a^
	CR-3	(3.9±0.3)^a^	(7.2±0.2)^a^	(12.3±0.2)^a^	(25.7±0.5)^a^
	CR-4	(2.1±0.2)^b^	(4.4±0.3)^b^	(7.9±0.3)^b^	(17.1±0.4)^b^
	CR-5	(2.5±0.3)^b^	(4.9±0.2)^b^	(9.3±0.3)^b^	(20.5±0.4)^a^
	CR-6	(2.5±0.2)^b^	(5.4±0.3)^b^	(10.7±0.4)^ab^	(21.8±0.2)^a^
	CR-7	(2.6±0.2)^b^	(5.7±0.3)^ab^	(12.4±0.2)^a^	(24.2±0.3)^a^
	CR-8	(3.7±0.3)^a^	(7.5±0.2)^a^	(13.4±0.3)^a^	(25.4±0.2)^a^
Peroxide value/(mmolof O_2_per kg of lipid)	CR-1	(0.28±0.04)^a^	(0.65±0.02)^a^	(0.87±0.04)^a^	(1.58±0.07)^a^
	CR-2	(0.25±0.03)^a^	(0.59±0.03)^a^	(0.91±0.06)^a^	(1.34±0.08)^a^
	CR-3	(0.34±0.03)^a^	(0.61±0.02)^a^	(0.94±0.03)^a^	(1.49±0.05)^a^
	CR-4	(0.25±0.01)^a^	(0.31±0.01)^b^	(0.63±0.07)^b^	(1.15±0.04)^b^
	CR-5	(0.26±0.03)^a^	(0.42±0.01)^b^	(0.74±0.02)^b^	(1.26±0.06)^ab^
	CR-6	(0.30±0.02)^a^	(0.41±0.02)^b^	(0.69±0.05)^b^	(1.29±0.05)^ab^
	CR-7	(0.21±0.02)^a^	(0.40±0.04)^b^	(0.75±0.03)^b^	(1.45±0.03)^a^
	CR-8	(0.27±0.03)^a^	(0.58±0.03)^a^	(0.92±0.08)^a^	(1.32±0.04)^a^

Results are expressed as mean value±standard deviation. Different letters in superscript indicate significant difference between the samples in the same row. Sample abbreviations are given in Table 1

At the beginning of the study, we found low peroxide values (less than 1.00 mmol of O_2_ per kg of lipid) in our sausages. Lorenzo *et al*. (*43*) state that the starter culture used in fermented sausages inhibits the formation of peroxide value during storage, while Falowo *et al.* (*44*) indicate that slower lipid spoilage in fermented sausages results from a decrease of moisture and the denaturation of enzymes in the meat during storage. In our study, the highest peroxide value was found in control samples of cold-smoked pork sausages, and the lowest in the samples with taxifolin and starter culture. Thus, the changes in peroxide values in fermented sausages indicate that taxifolin effectively inhibits chain reactions occurring in lipid peroxidation. These results coincide with the findings of Semenova *et al.* (*45*), who stated that the taxifolin even at the minimal mass fraction of 0.001% inhibits the oxidation of the lipid fraction in the minced meat, because it reduces peroxide value by 58.60% compared to the minced meat produced by the traditional recipe. Bakalivanova and Kaloyanov (*46*) also stated that taxifolin has a beneficial effect on lipid peroxidase and is suitable for use in sausage production as an antioxidant.

On the first day of the study, the lowest acid value (Table 4) was determined in batches CR-4, CR-5, CR-6 and CR-7 ((2.1±0.2), (2.5±0.3), (2.4±0.2) and (2.6±0.2) mg NaOH per kg of lipid, respectively) (p<0.05). Gonzales *et al.* (*16*) state that flavonoids are able to bind metals that are capable of catalysing many biological processes such as fat hydrolysis. Topal *et al.* (*2*) confirmed these results; they also found that taxifolin can bind free radicals and metal ions. At the end of the study (day 181), the significantly lower acid value was determined only in batch CR-4 ((17.1±0.4) mg/kg) (p<0.05). In the samples, a strong negative correlation was found between pH and peroxide value (R=-0.831, p<0.05) between pH and acid value (-0.874, p<0.05) (data not shown). We also found that the duration of storage influenced acid and peroxide values (p<0.05).

### Evaluation of colour prameters of cold-smoked pork sausages

We determined that the colour parameters (L*, a* and b*) of all preparations and final meat products were significantly (p<0.05) influenced by the used taxifolin and starter cultures and storage time (Table 5). Some reports indicate that changes in the colour of fermented sausage are influenced only by maturation time, but not by starter cultures (*47*). The redness (a*) of investigated cold-smoked sausages during storage decreased in all samples, but significantly only in samples CR-5, CR-6 and CR-7 from the 33rd day of investigation (p<0.05).

**Table 5:** Effect of taxifolin and commercial starter cultures on the colour of cold-smoked pork sausages during storage

Colour	Sample	*t*(storage)/day			
1	33	128	181
L*	CR-1	(36.6±0.3)^b^	(43.4±0.4)^a^	(39.9±0.0)^a^	(39.6±0.1)^a^
	CR-2	(40.1±0.7)^a^	(45.8±0.0)^b^	(45.1±0.9)^b^	(39.6±0.1)^a^
	CR-3	(35.3±0.3)^b^	(43.1±0.5)^a^	(41.1±0.1)^a^	(40.1±0.1)^a^
	CR-4	(38.9±0.5)^a^	(44.4±0.3)^a^	(43.0±2.7)^a^	(38.4±0.2)^a^
	CR-5	(39.9±0.4)^a^	(47.6±0.1)^b^	(45.6±0.3) ^b^	(46.7±0.0)^b^
	CR-6	(40.4±0.7)^a^	(48.1±0.1)^b^	(46.8±0.0)^b^	(43.2±0.3)^b^
	CR-7	(32.2±0.4)^b^	(41.2±0.4)^a^	(41.0±0.3)^a^	(39.3±0.1)^a^
	CR-8	(33.8±0.6)^b^	(40.6±0.1)^a^	(40.0±0.1)^a^	(38.4±0.1)^a^
a*	CR-1	(15.7±0.5)^a^	(25.9±0.4)^b^	(16.4±0.0)^b^	(14.8±0.0)^a^
	CR-2	(16.1±0.4)^ab^	(23.3±0.3)^b^	(15.7±0.6^b^	(15.5±0.1)^a^
	CR-3	(15.6±0.6)^a^	(18.0±0.5)^a^	(13.3±0.0)^a^	(11.8±0.0)^ab^
	CR-4	(16.0±0.3)^a^	(21.0±0.2)^b^	(14.1±2.6)^a^	(15.8±0.0)^a^
	CR-5	(18.3±0.5)^b^	(20.4±0.3)^b^	(15.9±0.1)^b^	(14.2±0.0)^a^
	CR-6	(17.5±0.6)^ab^	(20.6±0.6)^b^	(14.9±0.0)^a^	(14.7±0.1)^a^
	CR-7	(15.4±0.7)^a^	(20.4±0.1)^b^	(13.7±0.1)^a^	(10.9±0.1)^ab^
	CR-8	(14.4±0.7)^a^	(14.4±0.1)^a^	(12.8±0.0)^a^	(8.4±0.1)^b^
b*	CR-1	(13.0±0.2)^a^	(19.5±0.4)^a^	(8.9±0.0)^a^	(7.4±0.1)^a^
	CR-2	(13.0±0.2)^a^	(20.9±0.2)^a^	(10.0±0.4)^a^	(9.1±0.1)^a^
	CR-3	(11.7±0.6)^a^	(13.0±0.3)^a^	(7.4±0.0)^a^	(6.2±0.1)^a^
	CR-4	(12.5±0.4)^a^	(15.7±0.2)^a^	(10.6±1.3)^a^	(8.7±0.0)^a^
	CR-5	(13.0±0.6)^a^	(16.5±0.0)^a^	(9.3±0.0)^a^	(9.2±0.0)^a^
	CR-6	(13.2±0.5)^a^	(15.7±0.5)^a^	(8.5±0.0)^a^	(8.2±0.0)^a^
	CR-7	(10.9±0.2)^a^	(19.9±0.3)^a^	(10.6±0.1)^a^	(7.1±0.0)^a^
	CR-8	(11.9±0.3)^a^	(18.0±0.1)^a^	(11.3±0.0)^a^	(10.1±0.1)^a^

Results are expressed as mean value±standard deviation. Different letters in superscript indicate significant differences between the samples in the same row. Sample abbreviations are given in Table 1

## CONCLUSIONS

In this study, we evaluated the effect of taxifolin on the microbiological profile of cold-smoked pork sausages, as well as their acidity, biogenic amine content, and colour parameters. The stability and antioxidant activity of taxifolin were also evaluated. We found that taxifolin increased the acidity of cold-smoked pork sausages during storage, with lower pH values found in samples CR-3 and CR-6. Taxifolin had an inhibitory effect on mould and yeast, and inhibited fat peroxidation processes, leading to lower acid values. A negative correlation between the pH and acid value was also found. Taxifolin and a commercial starter culture with *Pediococcus pentosaceus* and *Staphylococcus xylosus* could reduce the accumulation of total biogenic amine values, and also stabilize and slow down the rate of lipolysis and effectively inhibit the processes of lipid peroxidation. On the other hand, taxifolin causes the colour to fade, which could be unfavourable effect, and modification of sausage fermentation would be needed. Taxifolin should be studied in detail to improve the food processes and provide maximum beneficial health effects to the consumers with optimum nutritional and functional properties. It is very important to continue working with different meat products and taxifolin to better understand the effect of interactions of different compounds. The outcome of this study can help develop new fermented meat products with beneficial health aspects.
